# *Spatholobus suberectus* Column Extract Inhibits Estrogen Receptor Positive Breast Cancer via Suppressing ER MAPK PI3K/AKT Pathway

**DOI:** 10.1155/2016/2934340

**Published:** 2016-12-21

**Authors:** Jia-Qi Sun, Gan-Lin Zhang, Yi Zhang, Nan Nan, Xu Sun, Ming-Wei Yu, Hong Wang, Jin-Ping Li, Xiao-Min Wang

**Affiliations:** ^1^Department of Oncology, Beijing Hospital of Traditional Chinese Medicine Affiliated to Capital Medical University, Beijing, China; ^2^Beijing Institute of Traditional Chinese Medicine, Beijing Hospital of Traditional Chinese Medicine Affiliated to Capital Medical University, Beijing, China; ^3^Department of Medical Biochemistry and Microbiology, Uppsala University, 75123 Uppsala, Sweden

## Abstract

Although Chinese herbal compounds have long been alternatively applied for cancer treatment in China, their treatment effects have not been sufficiently investigated. The Chinese herb* Spatholobus suberectus* is commonly prescribed to cancer patients. HPLC analysis has shown that the main components of* Spatholobus suberectus* are flavonoids that can be classified as phytoestrogens, having a structure similar to estrogen. This study was designed to investigate the effects of* Spatholobus suberectus* column extract (SSCE) on the estrogen receptor-positive (ER+) breast cancer cell line MCF-7 and its possible molecular mechanism. In our study, MTT assay was performed to evaluate cell viability. The results show that SSCE (80, 160, and 320 *μ*g/ml) significantly decreased the viability of MCF-7 cells. SSCE also triggered apoptosis, arrested the cell cycle at the G0/G1 phase, and inhibited cell migration. A dual-luciferase reporter system showed that SSCE suppressed intranuclear p-ER activity; Western blot analysis confirmed the repressed expression of phosphorylated-ER alpha (p-ER*α*), ERK1/2, p-ERK1/2, AKT, p-AKT, p-mTOR, PI3K, and p-PI3K, indicating that SSCE suppressed the MAPK PI3K/AKT signaling pathway. Collectively, our results suggest that SSCE causes apoptosis, an arrest in the G0/G1 phase, and a decrease in migration in ER+ MCF-7 cells via hypoactivity of the ER and suppression of the MAPK PI3K/AKT pathway.

## 1. Introduction

Breast cancer is the most common malignancy and the leading cause of cancer-related morbidity and mortality among women worldwide [1]. Endocrine therapy represents a major treatment in all settings of the disease for estrogen receptor-positive breast cancers, which account for approximately 70% of mammary malignancies [2]. In this subgroup of breast cancers, endocrine therapies are effective both in the adjuvant and in the recurrent settings; however, resistance remains a major issue [3].

*Spatholobus suberectus* Dunn, belonging to the legume family (Fabaceae), is called “chicken blood vines” in China due to its red juice, similar in appearance to blood, that flows out when the vine is injured [4]. It has been used to treat a variety of diseases such as anemia [5], menoxenia, and rheumatism in traditional Chinese medicine [6, 7]. The Taiwanese National Health Insurance Research Database showed that* Spatholobus suberectus* is the 8th most common single herb in adjunctive Chinese herbal medicine therapy for chronic myeloid leukemia patients, and Chinese herbal medicine therapy improves the survival of patients with chronic myeloid leukemia [8]. A few studies have been conducted reporting various types of flavonoids as the principal characteristic components in this herb [9]. Flavonoids are phytoestrogens, which have a similar structure to estrogen and can bind to estrogen receptors.

As an alternative therapy, traditional Chinese herb medicine is becoming accepted by more and more patients for controlling cancer [10], especially in the population who cannot tolerate increasingly intense conventional chemotherapy. Spatholobi Caulis is wildly used by mammary tumor patients in China; however, to date, its treatment effects and potential mechanisms have not been sufficiently investigated. It is of great importance to elucidate the effective mechanisms to support the clinical use of Spatholobi Caulis in ER-positive breast cancer patients. Our present research was designed to examine the effects of the extracts from* Spatholobus suberectus* (SSCE) on the functions of ER+ MCF-7 cells. The results show that SSCE inhibited cell growth via suppression of the MAPK PI3K/AKT pathway.

## 2. Material and Methods

### 2.1. Preparation of SSCE

*Spatholobus suberectus* column extract (SSCE) was prepared by the Beijing Institute of traditional Chinese Medicine.* Spatholobus suberectus* was obtained from Beijing Xinglin Pharmaceutical Co., Ltd. (place of production: Yunnan, Lot number: 11062601).* Spatholobus suberectus* (100 g) was extracted in 80% ethanol (500 ml) at room temperature over night followed by 2 h heating-refluxing extraction three times. After cooling down to room temperature, the mixture was filtered through a 180-mesh sieve, and the solvent was removed. The crude extract of* Spatholobus suberectus* was dissolved in deionized water and centrifuged (3500 r min^−1^, 20 min). The supernatant was collected and analyzed on a polyamide resin column (Φ1 cm, column height 11 cm). The column was eluted with deionized water and the elute was extracted with three passes of diethyl ether (10, 10, and 5 ml) and pooled. The ether was evaporated and 276 mg of SSCE was obtained. The chemical composition of SSCE was determined by HPLC analysis (Beijing Institute of Traditional Chinese Medicine) as summarized in Table 1.

### 2.2. Chemicals, Reagents, and Antibodies

Bax and p-ER*β* were purchased from Abcam (Cambridge, MA, USA). Other antibodies were all purchased from Cell Signaling Technology (Danvers, MA, USA). Cell culture medium (DMEM), penicillin-streptomycin, and fetal bovine serum (FBS) were purchased from Gibco (NY, USA). The plasmid was purchased from Promega. The dual-luciferase assay kit was purchased from Vigorous (Beijing, China). All primers were synthesized by Invitrogen™, Thermo Fisher Scientific.

### 2.3. Cell Culture

Human ER+ breast cancer cells (MCF-7) were obtained from The Basic Medical Research Institute of Medical Sciences Chinese cell resource center. The cells were cultured in high glucose DMEM medium supplemented with 10% fetal bovine serum (FBS) and 1% penicillin-streptomycin and incubated at 37°C in a humidified atmosphere with 5% CO_2_. The media was changed to DMEM without phenol red (Gibco number 31053-028) and 10% charcoal-stripped fetal bovine serum (Gibco number 12676-029) with 1% penicillin-streptomycin 24 h prior to the experiments.

### 2.4. Cell Viability Assay

MTT assay was performed to quantify cell viability. MCF-7 cells were seeded into 96-well plates (2 × 10^3^ cells per well) for 24 h and then exposed to various concentrations of E2 (10^−4^, 10^−5^, 10^−6^, 10^−7^, 10^−8^, 10^−9^, 10^−10^, 10^−11^, and 10^−12^ M), SSCE (0, 40, 80, 160, and 320 *μ*g/ml), and E2 at 10^−8 ^M coincubated with SSCE (0, 40, 80, 160, and 320 *μ*g/ml). After 24, 48, and 72 h, the cells were incubated with 3-[4,5-dimethylthiazol-2-yl]-2,5-diphenyl tetrazolium bromide (MTT) at 5 mg/ml and 10 *μ*l/well for 4 h. The culture medium was then discarded, and cells were lysed in DMSO with gentle shaking for 15 sec. The optical density (OD) values were measured at 570 nm using a Multiskan Spectrum (Thermo Scientific, USA). Experiments were performed in triplicate.

### 2.5. Flow Cytometry Assay

Apoptosis was demonstrated and quantified by flow cytometry analysis using an apoptosis kit (Cat. number KGA7073 Nanjing KeyGen Biotech Co.). MCF-7 cells were seeded into 6-well plates (2 × 10^5^/well). After treatment with SSCE (0, 160 *μ*g/ml) for 24 h, MCF-7 cells were trypsinized by trypsin without EDTA, centrifuged at 1200 r for 3 min, washed twice with PBS, and stained with Annexin V-fluorescein isothiocyanate (FITC) and propidium iodide (PI) according to the manufacturer's instructions. The cells were then filtered with a 300-mesh filter. Apoptotic cells were detected with a Beckman flow cytometry system (EPICS XL, Beckman) and analyzed with FlowJo 7.6.1 software (Tree Star). Each assay included at least 10,000 gated events. The early apoptotic cells are Annexin V-FITC positive and PI negative, while late apoptotic cells are Annexin V-FITC and PI double-positive. Experiments were performed in triplicate.

The cell cycle was observed and quantified using propidium iodide (PI) staining for DNA by flow cytometry analysis. MCF-7 cells were seeded into 6-well plates (2 × 10^5^/well) with phenol red-free DMEM media and charcoal-stripped fetal bovine serum. In addition, the cells were treated with 10^−8^ M E2 (as control), 10^−8^ M E2 and SSCE at the indicated concentration (0, 160, and 320 *μ*g/ml), 10^−8^ M E2 and 10^−4 ^M ICI182780, and DMEM without E2 for 24 h. The cells were harvested and washed twice with ice-cold PBS. Cells were fixed in 70% ethanol overnight and washed once with PBS. After centrifugation at 1200 r for 3 min and resuspension in 400 *μ*l PBS with 50 *μ*g/ml propidium iodide (PI) and 20 *μ*g/ml RNase A, the cells were incubated for 30 min at 37°C in the dark and filtered with a 300-mesh filter. PI fluorescence was detected using a Beckman flow cytometry system (EPICS XL, Beckman) and analyzed with FlowJo 7.6.1 software (Tree Star). Each assay included at least 10,000 gated events. Experiments were performed in triplicate.

### 2.6. Wound Healing Assays

Cells were seeded in 6-well tissue culture plates at 5 × 10^5^ per well. When the cells grew into a monolayer, a scratch was created using a sterile p200 pipette. The scratch was created vertically in the middle of each well. The medium was removed, and the detached cells were removed by rinsing each well with 1 ml PBS. Formation of the wound was confirmed by inverted light microscopy. After addition of phenol red-free serum-free media, the cells were then incubated with SSCE (160 *μ*g/ml), estradiol (10^−8 ^M), and SSCE (160 *μ*g/ml) + estradiol (10^−8 ^M) and monitored by microscopic observation. Two vertical lines on each side of the scratch and one horizontal line, separating the wound in half, were placed with an indelible marker on the outside bottom of each well to ensure that the same field was identified during subsequent image acquisition. These markings served as reference points for photographic documentation. The change in the wound surface area was compared among groups over time. Digital photographic images were obtained at 0 h and 24 h using a motorized inverted microscope. Following the acquisition of all images, the surface area of each scratch was measured and outlined by two independent observers (blinded to the group situation) using Adobe Photoshop software. The surface area of each wounded region of the cell monolayer was then transformed into a square of equal surface area, and the linear mean length of each square was compared among groups. The rate of closure was quantified and compared between all groups for statistical analysis. Three replicate wells were used for each experimental condition. Experiments were performed in triplicate.

### 2.7. Western Blot Assay

MCF-7 cells were exposed to SSCE at the indicated concentration (0, 80, 160, and 320 *μ*g/ml) and incubated for 24 h. The cells were washed once with PBS and collected. The cells were lysed for 30 min in lysis buffer (Beyotime, P0013K) containing cOmplete EDTA-free protease inhibitor (Roche, Cat. number 04693132001; 1 tablet suspended in 1 ml ultrapure water and added 1 : 25 to the lysis buffer) and PhosSTOP (Roche, Cat. number 04906845001; 1 tablet suspended in 1 ml ultrapure water and added 1 : 10 to lysis buffer). The lysates were subjected to shaking once every five min for the 30 min incubation. Then, the lysates were centrifuged at 12000 r for 15 min. The supernatants were transferred into new Eppendorf tubes, and the total protein was quantified. The samples were separated in a 10% gradient SDS-PAGE (the proteins were separated in an 8% gradient SDS-PAGE for the detection of mTOR and p-mTOR) and transferred onto nitrocellulose membranes. The membrane was blocked using a blocking buffer (5% milk, 20 mmol/l Tris-HCl, pH 7.6, 150 mmol/l NaCl, and 0.1% Tween 20) at room temperature for 1.5 h. The membranes were then incubated with antibodies against PCNA, ERK1/2, p-ERK1/2, PI3K, p-PI3K, AKT, p-AKT, mTOR, p-mTOR, cytochrome C, Bax, Bcl-2, phosphorylated ER*α* (Ser118), phosphorylated ER*β* (Ser105), and *β*-actin, followed by incubation with secondary antibodies. Densitometric analysis of the immunoblots was performed using an Odyssey infrared imaging system (LI-COR Biosciences Company, USA). Experiments were performed in triplicate.

### 2.8. Dual-Luciferase Assay

MCF-7 cells were seeded into 24-well plates at the exponential phase of growth (approximately 2 × 10^5^/well) and cultured for 18–24 h. Then, the serum containing medium was replaced by OPTI-MEM for transfection using Lipo2000 mixed with different plasmids, including 3X ERE TATA luc, pcDNA Flag ER beta, pEGFP-C1-ER alpha, pFLAG-CMV2, pEGFP-C1, and the Firefly luciferase plasmid: pGL3-Basic Vector and pRL-TK Vector at a 2000 : 1 ratio (1 *μ*l lipo2000 : 0.5 *μ*g plasmid). After 5 h, the cells were changed from the transfection medium to the normal medium with serum. The cells were cultured for 24 h before addition of SSCE. After a12 h incubation, the cells were collected, counted, and seeded onto 96-well plates with each sample in 3 wells. The luciferase activity was detected following the instructions in the dual-luciferase assay kit (Vigorous, Cat # T002). Briefly, the cells were washed with PBS, lysed in 30 *μ*l/well 1x Universal Lysis Buffer (ULB), gently shaken for 10 min, and centrifuged at 1200 r for 0.5 min. The supernatants were placed in new Eppendorf tubes for luminescence detection. To detect the Firefly luciferase and the sea kidney luciferase, specific steps were carried out according to kit instructions.

### 2.9. Statistical Analysis

Data for cell proliferation were converted into percentages, where the values for the control condition were formulated as 100%. All data were presented in comparison to controls. All experiments were performed in triplicate. Statistical significance of the difference was calculated using Student's *t*-test for paired data with the level of significance selected at *P* < 0.05.

## 3. Results

**Figure 1:**
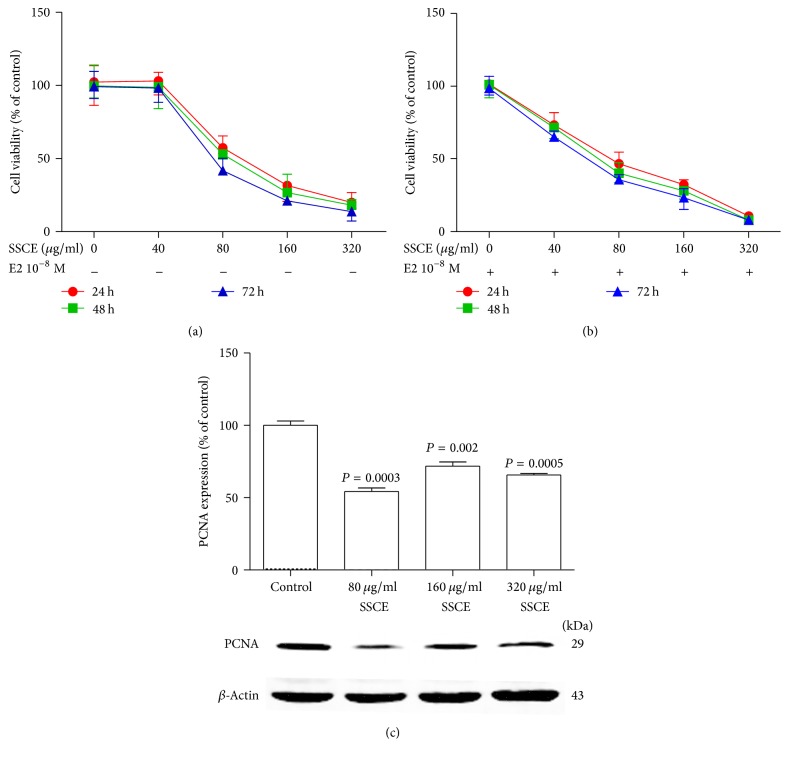
Inhibition of the viability of MCF-7 cells with SSCE treatment. (a) SSCE treatment in the absence of 17*β*-estradiol (E2) inhibited the proliferation of MCF-7 cells. The IC50 of SSCE treated for 24 h, 48 h, and 72 h was 109 *μ*g/ml, 102 *μ*g/ml, and 83 *μ*g/ml, respectively. (b) Addition of 10^−8 ^M E2 promoted the effect of SSCE. The IC50 of SSCE in the presence of 10^−8 ^M E2 was 81 *μ*g/ml, 68 *μ*g/ml, and 60 *μ*g/ml for a 24 h, 48 h, and 72 h incubation, respectively. (c) The proliferation-related protein (PCNA) level was decreased upon treatment with SSCE (80 *μ*g/ml, 160 *μ*g/ml, and 320 *μ*g/ml) for 24 h compared to control.

SSCE inhibited the vitality of breast cancer MCF-7 cells both in the presence and in the absence of E2. We first examined the effect of different concentrations of E2 on the viability of ER+ MCF-7 cells using MTT assay and found that, at the concentration of 10^−8 ^M E2, the cells exhibited the highest viability with approximately 35% proliferation (data not shown). SSCE inhibited the proliferation of the MCF-7 cells in a dose-dependent manner (Figure 1(a)) with an estimated IC50 value of 109 *μ*g/ml after 24 h incubation. In the presence of E2 at the concentration of 10^−8 ^M, the effect of SSCE was enhanced (Figure 1(b)) with an IC50 value of 81 *μ*g/ml after 24 h incubation. The inhibitory effect of SSCE was further demonstrated by the reduced level of the proliferation-related protein PCNA (Figure 1(c)).

### 3.1. SSCE Triggered Apoptosis of MCF-7 Cells

**Figure 2:**
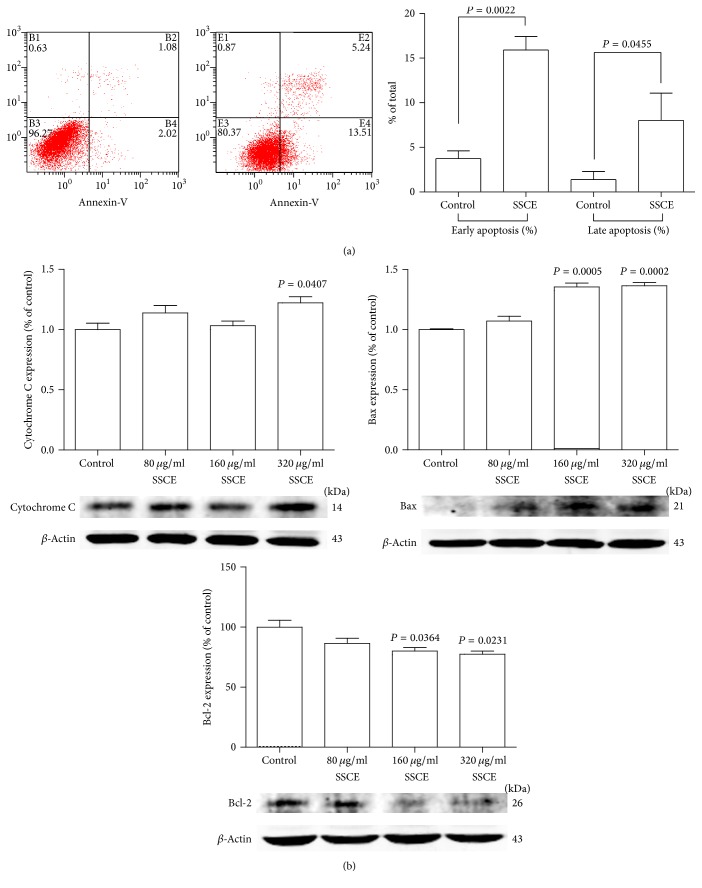
(a) SSCE (160 *μ*g/ml) treatment for 24 h induced apoptosis of MCF-7 cells with a significant increase in the proportion of both early and late apoptotic cells analyzed by FACS. (b) Western blot analysis and quantification of the band intensity show that after SSCE (80 *μ*g/ml, 160 *μ*g/ml, and 320 *μ*g/ml) treatment for 24 h, the expression levels of apoptosis-related proteins cytochrome C and Bax were elevated, and Bcl-2 expression was suppressed.

Apoptotic cells identified by a flow cytometry assay were significantly increased in the MCF-7 cells treated with SSCE at the concentration of 160 *μ*g/ml for 24 h. Both early apoptotic cells and late apoptotic cells were increased and the differences compared to the control group were statistically significant (Figure 2(a)). Then, we assessed the apoptotic related proteins cytochrome C, Bax, and Bcl-2 by Western blot analysis. It showed that SSCE (80 *μ*g/ml, 160 *μ*g/ml, and 320 *μ*g/ml) upregulated the levels of cytochrome C and Bax and downregulated the level of Bcl-2 (Figure 2(b)).

### 3.2. SSCE Triggered a G0/G1 Phase Arrest of MCF-7 Cells

**Figure 3:**
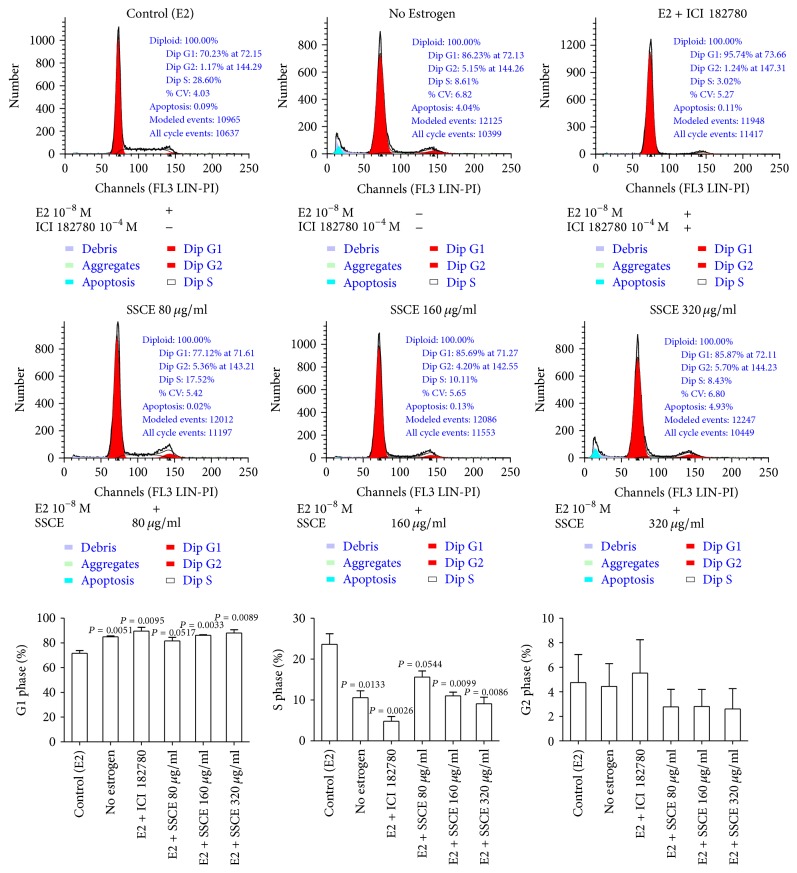
The control groups included MCF-7 cells cultured in DMEM with 10^−8 ^M E2 and cells cultured in the absence of E2. When treated with SSCE (80 *μ*g/ml, 160 *μ*g/ml, and 320 *μ*g/ml) in the presence of 10^−8 ^M E2, the proportion of cells in the G1 phase of the cell cycle was exacerbated. The proportion of cells in the S phase was reduced in SSCE treated groups. There was no difference in the proportion of cells in the G2 phase between these groups. The cells were cultured in phenol red-free DMEM with charcoal-stripped FBS.

We analyzed the cell cycle of the MCF-7 cells cultured under different concentrations of SSCE (80 *μ*g/ml, 160 *μ*g/ml, and 320 *μ*g/ml) in the presence of 10^−8^ M E2 for 24 h. The flow cytometry results showed that SSCE treatment induced a dose-dependent accumulation of cells arresting in the G0/G1 phase, with a reduction in the proportion of cells in the S phase. Similar results were obtained for the cells cultured in the presence of ICI182780 (10^−4^ M) + E2 (10^−8^ M) or in the absence of E2 (the no-estrogen cultured group) (Figure 3).

### 3.3. SSCE Inhibited the Migration of MCF-7 Cells

**Figure 4:**
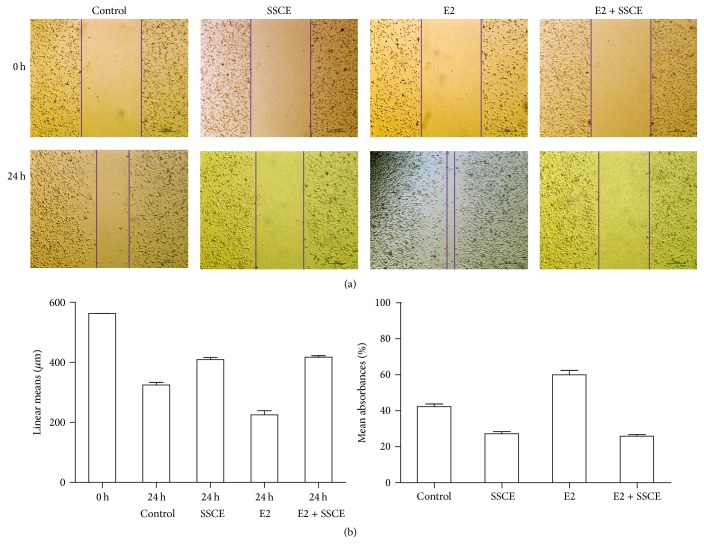
Wound healing assays to assess the effect of SSCE on migration ability. MCF-7 cells were cultured in a 6-well plate to approximately 90% confluency. A wound was generated by a scratch in the middle of the plate using a pipette. The wound closure was measured upon treatment with E2 (10^−8 ^M) and SSCE (160 mg/ml) alone or E2 (10^−8 ^M) + SSCE (160 mg/ml) for 24 h. (a) Representative images show the wound at 0 and 24 h. The migration of MCF-7 cells was promoted by E2 and inhibited by SSCE both in the presence and absence of E2. (b) The linear mean length and the confidence interval for each wounded area 24 h after the scratch are presented and compared to 0 h. The linear mean length of the control and the cells treated with SSCE and SSCE + E2 were significantly reduced compared to that in the other groups (*P* < 0.001). There was a significant difference noted in the linear means between the control and the E2-treated cells (*P* < 0.001). The rate of closure with the condition interval for each group 24 h after scratch is presented. The rates of closure of the SSCE group and SSCE + E2 group were significantly lower than that in the other groups.

Wound healing assays were used to assess the effect of SSCE on the invasion and migration ability of MCF-7 cells. The images of the scratches were documented at 0 and 24 h after the wounding (Figure 4(a)), and the closure of the wound was measured (Figure 4(b)). The healing assay indicated that, compared to the MCF-7 culture with no estrogen, E_2_ (10^−8 ^M) enhanced the invasion and migration of MCF-7 cells and SSCE (160 *μ*g/ml) significantly suppressed the invasion and migration of MCF-7 cells both in the presence and in the absence of E_2_ (10^−8 ^M) (Figure 4).

### 3.4. SSCE Inhibited the Activity of the Estrogen Receptor (ERE)

**Figure 5:**
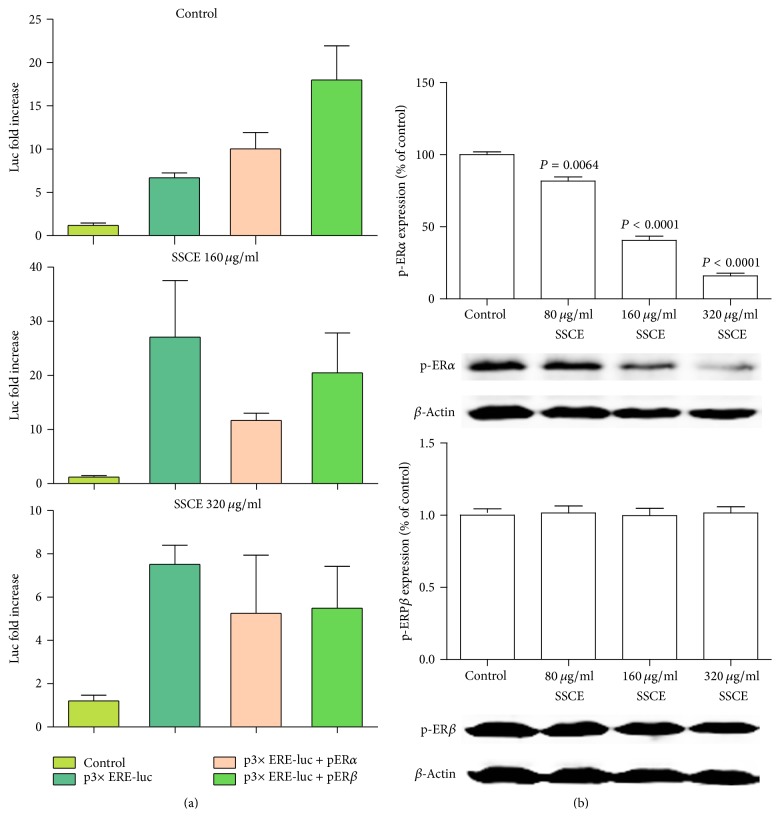
(a) The activity of nucleus ERE, p-ER*α*, and p-ER*β* in MCF-7 cells cultured without estrogen detected by a double-fluorescence enzyme reporting system is shown. Relative to ERE, the activation of p-ER*α* and p-ER*β* in the control group was significantly higher than that in MCF-7 cells cultured with SSCE (160 *μ*g/ml and 320 *μ*g/ml) for 12 h. (b) p-ER*α* and p-ER*β* protein levels after treatment with SSCE (80 *μ*g/ml, 160 *μ*g/ml, and 320 *μ*g/ml) cultured for 24 h were detected by Western blot. SSCE inhibited p-ER*α* expression in a concentration-dependent manner. However, SSCE did not affect the expression of p-ER*β*.

The cells were transiently transfected with the constructs as described in the Material and Methods section. The activity of intranuclear p-ER activity was detected using a dual-luciferase reporter system. The activity of p-ER*α* and p-Er*β* in MCF-7 cells cultivated in the medium without estrogen is shown in Figure 5(a). The luciferase activity in p-ER*α* and p-ER*β* is higher than that in the ERE-transfected cells. SSCE (80 *μ*g/ml, 160 *μ*g/ml, or 320 *μ*g/ml) treatment for 12 h attenuated the luciferase activity in the p-ER*α*- and p-ER*β*-transfected cells, especially in the group treated with 160 *μ*g/ml SSCE (Figure 5(a)).

Expression of the receptors was analyzed by Western blot. The control group exhibited a strong phosphoestrogen receptor *α* (Ser118) protein band that was reduced in the cells cultured in the presence of SSCE (80 *μ*g/ml, 160 *μ*g/ml, or 320 *μ*g/ml) for 24 h. Inhibition of expression of the receptors by SSCE was dose dependent (Figure 5(b)). In contrast, SSCE treatment did not affect the expression of phosphoestrogen receptor *β* (Ser105) (Figure 5(b)).

### 3.5. SSCE Regulated the MAPK Signaling Pathway

**Figure 6:**
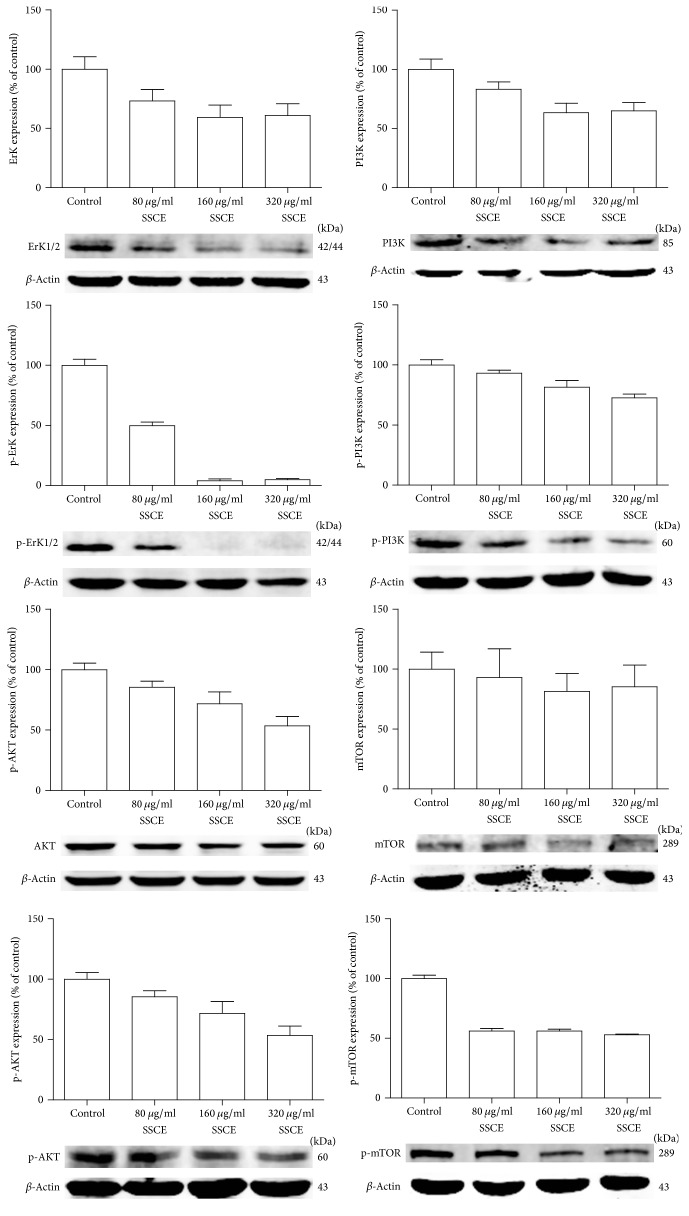
Western blot analysis showing that expression levels of ERK1/2, p-ERK1/2, AKT, p-AKT, PI3K, p-PI3K, and p-mTOR were significantly inhibited in the MCF-7 cells treated with SSCE (80 *μ*g/ml, 160 *μ*g/ml, and 320 *μ*g/ml) in a concentration-dependent manner. The expression of mTOR had no obvious association with the SSCE concentration.

Finally, we investigated the typical protein signaling events in the MAPK PI3K/AKT pathway by Western blot to determine whether SSCE affected the protein expression. The expression levels of ERK1/2, p-ERK1/2, AKT, p-AKT, p-mTOR, PI3K, and p-PI3K were significantly reduced in the MCF-7 cells treated with SSCE in a concentration-dependent manner. In comparison, there was no significant difference in the expression of mTOR between the different concentrations of SSCE (Figure 6).

## 4. Discussion

Earlier studies reported that Spatholobi Caulis extract inhibited proliferation of KB, K562, and HL60 cells with an IC50 value of 17.6, 8.3, and 9.7 mg/ml, respectively [11]. Spatholobi Caulis was also demonstrated to inhibit platelet aggregation induced by B16BL6 melanoma cells with an IC50 of 50 mg/ml. It also inhibited HT1080 cancer cell invasion through a Matrigel-coated filter with an IC50 of 25 mg/ml [12]. SSCE was also found to induce apoptosis via the caspase-dependent pathway in U937 cells [13]. In line with the previous reports, we found similar antiproliferation effect of Spatholobi Caulis extract (SSCE) in MCF-7 cells with an IC50 value of 109 *μ*g/ml. SSCE treatment resulted in inhibition of MCF-7 migration as observed in a scratch test, induced apoptosis, and arrested the cell cycle at the G0/G1 phase.

Estrogens are suggested to trigger breast cancer by stimulating cell proliferation through receptor-mediated processes and by way of their toxic metabolites [14, 15]. A 15-year randomized, placebo-controlled trial involving 161808 postmenopausal women revealed that long-term use of hormone replacement therapy (HRT) is associated with an increased risk of breast cancer [16]. Thus, steroidal was added to the list of known human carcinogens in the US in 2001 [17]. Spatholobi Caulis is wildly used for the treatment of ER+ mammary tumor patients in China. The main components of* Spatholobus suberectus* column extract (SSCE) from Spatholobi Caulis, analyzed by HPLC [18], include formononetin, genistein, calycosin, and daidzein, all of which are flavonoid phytoestrogens [18]. Phytoestrogens are biologically active compounds of plant origin that structurally mimic the mammalian steroid hormone 17*β*-estradiol (E2) and are mainly found in vegetables, fruits, and herbs [19]. Phytoestrogens bind with estrogen receptors and may modulate estrogen receptor activity [20, 21]. Studies have shown that women in the US have a three times higher risk for breast cancer than Asian women [22]. This difference has been partially associated with Asian diets that consist of a considerably large proportion of phytoestrogens [20, 21]. Some phytoestrogens can reduce the carcinogenic effect of bisphenol A by blocking the connection of bisphenol A and ER [23, 24]. Epidemiologic and experimental evidence suggest that phytoestrogens play a chemopreventive role in breast cancers. Our findings provided further biological evidence for the effect of phytoestrogen-type molecules in treatment of breast cancer.

**Figure 7:**
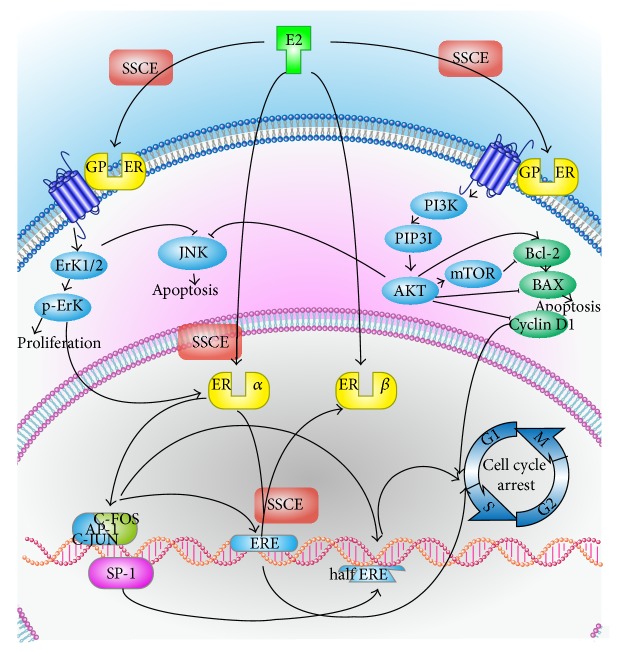
The classic mechanism for E2 action is E2 diffusing into the nucleus, binding to the nucleus estrogen receptor (ER), combining with ERE on DNA, starting transcription translation and inducing the cycle of multiplication in cancer cells. In addition, activated ER*α* can activate AP-1 and SP-1, which are transcription enhancers that can promote the transcription translation of ERE and enable incomplete ERE (half ERE) to transcribe and translate. SSCE may affect the activation of ER*α*, decreasing p-ER*α*, reducing the combination ability of ER and ERE, and stopping ERE from transcribing and translating. On the other hand, E2 can combine with GPER on the cell membrane, increase the expression of ERK, promote cell proliferation, activate the PI3K-AKT pathway, increase the activation of mTOR and the upregulation of Bcl-2 and cyclin D, downregulate Bax, reduce apoptosis, and start the cell cycle. Both ERK and AKT can inhibit JNK, reducing apoptosis. SSCE may downregulate the PI3K-AKT, ERK pathway by GPER, resulting in a downregulation of Bcl-2, upregulation of Bax, reduction in the inhibition of JNK, promotion of cell apoptosis, and downregulation of cyclin D, thereby blocking the cell cycle.

There are two main pathways by which E2 plays its role. In the classical pathway, E2 diffuses into the nucleus and binds to the ER. Once activated by E2, the ER is able to be phosphorylated and translocates into the nucleus where it binds to specific sequences of DNA known as estrogen response elements (ERE) [25]. The DNA receptor complex then recruits other proteins that are responsible for the transcription of downstream DNA into mRNA and finally protein that results in a change in cell function. In addition, activated ER also interacts with activator protein 1 (AP-1) and transcription factor specificity protein 1 (Sp-1) to promote transcription via several coactivators such as PELP1 [25]. AP-1 activity can be regulated by the ERK pathway. This results in activated c-jun and its downstream targets such as RACK1 and cyclin D1 [26]. Cyclin D1 is required for the progression through the G1 phase of the cell cycle to the S phase and reduced cyclin D1 expression results in G1 arrest [27]. c-Jun is overexpressed in MCF-7 cells [28]. c-Jun can also protect cells from apoptosis [27] (Figure 7).

The activity of nucleus p-ER*α* and p-ER*β* was suppressed by SSCE. SSCE may affect the activation of ER*α*, decreasing p-ER*α*. Inactivated ER cannot bind to ERE, preventing the transcription and translation of ERE and resulting in arrest of the cell cycle. It has been reported that phosphorylation of S105 ER*β* in breast cancer is associated with improved survival. Even in endocrine-resistant breast tumors, S105-ER*β* might be a useful additional prognostic marker [29]. Low S118 and high S167 ER-phosphorylation are associated with a disease-free state and overall survival [30]. In the same line, our results show that SSCE treatment attenuated p-ER*α* (S118) expression but did not affect expression of p-ER*β* (S105).

The second pathway of E2 is through binding to G protein-coupled ER on the cell membrane [31], increasing expression activity of ERK and activating the PI3K-AKT pathway [32]. As the mitogen-activated protein kinase (MAPK) cascades are major signaling transduction molecules in apoptosis [33] and MAPK signaling pathways have been identified as chemotherapeutic targets for sensitizing cancer cells to apoptosis [34], the reduction of ERK and p-ERK protein expression by SSCE treatment may have contributed to the inhibitory effect on the proliferation of the tumor cells. Further, the PI3K/AKT signal transduction pathway plays a pivotal role in cell survival and prevents cancer cells from apoptosis during tumorigenesis [35]. This is also in agreement with our findings that SSCE impaired the expression of AKT, p-AKT, PI3K, p-PI3K, and p-mTOR, repressing the PI3K/AKT pathway, downregulating Bcl-2, and upregulating Bax, which consequently induced cell cycle arrest and apoptosis. It appeared that SSCE downregulated the PI3K-AKT, ERK pathway by GPER; however, we cannot rule out whether SSCE has affected expression of other pathways, as it was reported that inhibition of ER signaling by tamoxifen enhanced HER2 expression [36].

## 5. Conclusion

In conclusion, our results showed that the* Spatholobus suberectus* column extract (SSCE) induced apoptotic cell cycle arrest of MCF-7 cells, inhibited activity of nucleus p-ER*α* and p-ER*β*, and downregulated the expression of p-ER*α*, which resulted in suppression of the MAPK PI3K/AKT pathway. The results indicate that the herbal extract inhibited the proliferation of ER-positive MCF-7 cells, contributing to the suppression of ER activity. Our data support the use of Spatholobi Caulis as a complementary medicine for management of breast cancer patients.

## Figures and Tables

**Table 1:** The components of SSCE detected by HPLC.

Peak	Components	Ratio in subfraction
1	Protocatechuic acid	0.704%
2	Unknown	1.183%
3	p-Hydroxybenzoic acid	0.486%
4	Epicatechin	0.927%
5	Puerarin	0.800%
6	Unknown	2.836%
7	Unknown	1.890%
8	Daidzein	9.504%
9	Glycyrrhizin	4.369%
10	Calycosin	4.555%
11	Unknown	5.340%
12	Genistein	7.204%
13	Formononetin	39.418%
14	Unknown	9.221%
15	Unknown	8.276%
16	Prunetin	3.288%
